# The (Gami)fictional Ego-Center: Projecting the Location of the Self Into an Avatar

**DOI:** 10.3389/fpsyg.2022.918688

**Published:** 2022-07-07

**Authors:** Maximilian A. Friehs, Sarah Schäfer, Christian Frings

**Affiliations:** ^1^UCD School of Psychology, University College Dublin, Dublin, Ireland; ^2^Lise Meitner Research Group Cognition and Plasticity, Max Planck Institute for Human Cognitive and Brain Sciences, Leipzig, Germany; ^3^Department of Cognitive Psychology and Methodology, University of Trier, Trier, Germany

**Keywords:** self, self-location, self-perception, self-prioritization, digital game, gamification, game design

## Abstract

A rich body of research suggests that self-associated stimuli are preferentially processed and therefore responses to such stimuli are typically faster and more accurate. In addition, people have an understanding of what they consider their “Self” and where it is located, namely near the head and upper torso—further boosting the processing of self-related stimuli if they are presented near the felt location of the self. We were interested in whether the same mechanism can be found when people transfer their “Self” into a static avatar. We investigated this in two studies with *N* = 33 and *N* = 39 young, healthy adults, respectively. Taken together, the results showed that (i) people indeed show enhanced processing for self-avatar-related stimuli and (ii) that self-associations are stronger if the to-be-associated stimuli are closer to the avatar’s upper torso—suggesting some kind of a projected location of the self in the avatar. This implies that attention is not equally distributed across the avatar. Beyond a theoretical level, this also has implications for practical use. For example, digital games opting for a non-traditional user interface where information is displayed on or in the direct vicinity of the character should take this effect into account when choosing which information to present where (i.e., present the most crucial piece of information close to the self-center of the avatar).

## Significance Statement

People prioritize self-relevant information and research shows that it is possible to transfer the Self into a previously neutral avatar. When a self-association with an avatar is built, there is a processing advantage for information that is closer to the ego center of the avatar. Since people are often interacting with or as avatars in games or applications, this result can inform design decisions.

## Introduction

The digital-games industry has grown dramatically in the last decade and it is predicted that, by 2023, over three billion people worldwide will play digital games, with players spending over $150 billion per year (Hawkins et al., 2013). Be it on mobile, consoles, or PCs: over half of all people with internet access play games. During the global COVID-19 pandemic, digital games have increasingly become the leisure activity of choice. For example, with no face-to-face contact possible, online games are a way to build trust, form friendships, and generally engage socially (Depping et al., 2016, 2018; Birk et al., 2017; Depping and Mandryk, 2017). As digital games moved beyond a mere niche leisure activity into a mainstream phenomenon for all ages, competition between games is high and game developers try to outdo their competitors by realizing the best possible user experience, keep their players engaged, and thus generate the most revenue (Birk et al., 2017; Alexandrovsky et al., 2019, 2021; Bowey et al., 2019).

An important aspect of games is the story and the story-based choices a player is confronted with; both can aid in transporting the player into the game world (Bowey and Mandryk, 2017; Bowey et al., 2019). Importantly, to experience the story, in most games, the player takes control of one or more avatar(s) and in multiplayer games, avatars from other players may be seen on screen. Understanding how players interact and identify themselves with avatars presents designers and researchers with unique questions and challenges. In that regard, subjective identification with the avatar has recently been assessed with the Player Identification Scale (PIS; Van Looy et al., 2012), and evidence suggests that increased avatar identification in turn increases motivation (Birk et al., 2016; Birk and Mandryk, 2018, 2019), which may enhance performance (Friehs et al., 2022). To put it differently, researchers assume that motivation increases based on the identification and self-association with the avatar, and the more an individual’s self-perception is associated with the avatar, the higher the motivation for an avatar-related task will be (see also Bessière et al., 2007).

An associated phenomenon has also been studied under the term *Proteus* effect (Yee and Bailenson, 2007). Named after the Greek god Proteus, this effect describes the influence of virtual self-representation on behavior and experiences. In their original work, Yee and Bailenson (2007) had participants, for example, take the role of a more or less conventionally attractive avatar in a virtual reality environment and measured the interpersonal distance. The authors found that the attractiveness of the embodied avatar was related to the interpersonal distance to a confederate in the study. The authors concluded that individuals tend to adjust their behavior to conform to their digital self-representation. Following in the footsteps of this research, Kocur et al. (2020a,2021) investigated the effect of virtual reality avatars’ athleticism on participants’ physical performance on cycling and isometric force tasks. Their results show that embodying an avatar associated with increased physical performance can enhance physical performance and decrease the subjective perception of exertion. The Proteus effect is argued to rely on similar mechanisms as body-ownership illusions, such as the rubber hand illusion (Botvinick and Cohen, 1998; Petkova and Ehrsson, 2008; Riemer et al., 2019). Taken together, it seems reasonable to assume that a more visceral connection between a person and their avatar is possible.

### Increasing the Self-Association via the Self-Prioritization Effect

While studies making use of the Proteus effect quiet literally aim to have an individual identify with the body of their avatar and body-ownership illusions try to have individuals perceive that something is part of their body, these effects do not specifically investigate the possibility of transferring the self-concept and, in a way, do not separate mind from body (Banakou et al., 2013, 2016; Kocur et al., 2020b). A useful tool to create a strong association between a formerly neutral stimulus (like, e.g., an avatar in a computer game) and the self is the so-called matching paradigm (Sui et al., 2012). This association is abstract and not directly linked to bodily perception. In this paradigm, participants are instructed to associate themselves with formerly neutral stimuli and after following this procedure, the newly self-associated stimuli are processed more efficiently—an effect termed Self-Prioritization Effect (SPE; see Sui et al., 2016; Schäfer et al., 2020). This paradigm has the key benefit of utilizing newly formed self-associations and thus conclusions about self-relevance effects can be drawn without any interference from formerly, long-term established and potentially confounded self-associations. To create self-associations, a participant is instructed to learn combinations of formerly neutral stimuli with either themselves or with non-self-relevant others (e.g., “You are the triangle. Someone else it the square”). Afterward, various combinations of the used labels and the stimuli are presented shortly one after the other and the participant is instructed to decide whether each combination matches the previously learned combinations or not (for more details, see Sui et al., 2012; Schäfer and Frings, 2019; Schäfer et al., 2020). In general, the SPE is depicted by faster and more accurate responses to self-relevant stimuli (Sui et al., 2012; Schäfer et al., 2015) and can thereby be seen as a measure of the strength of self-associations. Further, when the consequences of a body-ownership illusion can potentially be felt viscerally (e.g., when a rubber hand is struck and the individual moves back their own, unaffected hand in shock), the effects of the SPE are more subtle. The SPE is an effect that describes a prioritization in information processing that results in an advantage of self-associated stimuli in the range of, on average, 100–200 ms that is not consciously perceived by the individual. Importantly, this effect seems to be very robust as it has been demonstrated in different modalities (i.e., in vision, touch, and audition; Schäfer et al., 2016). Above that, it has been shown several times that various sorts of stimuli can be associated with the self. For example, complex concepts were prioritized once they were integrated (Schäfer et al., 2015), as well as amodal rhythms (Schäfer et al., 2020) and movements (Frings and Wentura, 2014).

Several studies already provided evidence that also avatars can be associated with the self and will then be prioritized (Mattan et al., 2015, 2017). Such self-associations with digital game characters are in line with studies that show that the personality factors predict avatar choices (Poeller et al., 2018, 2020, 2021) and players tend to rate their avatar more positive as compared to other avatars (e.g., Bessière et al., 2007).

### The Location of the Self

A large-scale survey by Limanowski and Hecht (2011) reported that people identify similar areas as the location of their self by demonstrating that self-location judgments were mainly centered around the upper torso or the head. Subsequent research provided additional support for the localization of the self around these areas (Starmans and Bloom, 2012; Alsmith and Longo, 2014). Similarly, when participants are asked to indicate when a cartoon fly was presented in front of the location of the self of a cartoon girl, the results not only confirmed previous results (i.e., location of the self at the upper torso or head) but extended them to non-human, and humanoid alien figures as well (Starmans and Bloom, 2012). This hypothesis was further strengthened experimentally in a study using the matching task to locate the self-center. In detail, the SPE was significantly larger, when the to-be-associated stimuli were presented close to the upper torso or head of the participant in comparison to when it was presented further away from the body (Schäfer et al., 2019). Taken together, two conclusions can be drawn based on those studies. First, the perceived location of the self was consistently measured close to the upper torso or head across samples and studies. Second, there is strong evidence that people are able to identify the location of the self in others or an avatar.

### The Current Study: Measuring Self-Projections Into an Avatar

To identify a potential self-location in an avatar, we run two experiments. We associated one avatar with the participant’s self and other avatars with others. Subsequently, we varied whether, during the matching task, the to-be-associated stimuli were presented close to the upper torso of the avatar (the “near” condition) or further away from it, specifically close to the legs (the “far” condition). We measured the SPE in dependence on the distance of the to-be-associated stimuli with the hypothesized location of the self in the avatar. Important to note is that the present study utilizes static avatars as opposed to more game-like, dynamic models. Further, the task itself does not allow for extensive avatar control and there are no character-dependent game mechanics such as character progression through a level-up system (Willumsen, 2018). The avatar in the present study only assumes the role of being representative of the player (in an abstract sense) or not.

We hypothesized that, as people tend to integrate stimuli more easily into their self-concept when those stimuli are perceived as being close to their upper torso and thereby close to the hypothesized location of the self (Schäfer et al., 2019), a similar effect should occur for an avatar after a strong association of this avatar with the self has been built. Specifically, we hypothesized the same data pattern as in Schäfer et al. (2019): a larger SPE when the to-be-associated stimuli are presented close to the upper torso (i.e., in the “near” condition) compared to when they are presented further away from it (i.e., in the “far” condition). If the location-specific SPE persists across experiments and participants, there is strong evidence for the robustness of such an effect.

The outcome of the study has implications for understanding if and how the power of the SPE can be harnessed in digital games. In a lot of games, especially in competitive ones, it is crucially important to design the most efficient user interface (UI) and to improve player performance. This is especially critical for games that choose to employ UIs where information about the avatar and the environment is displayed on or near the avatar (e.g., a health indicator is presented above the avatar head and not on a health bar in the corner of the screen).

## Experiment 1

**FIGURE 1:**
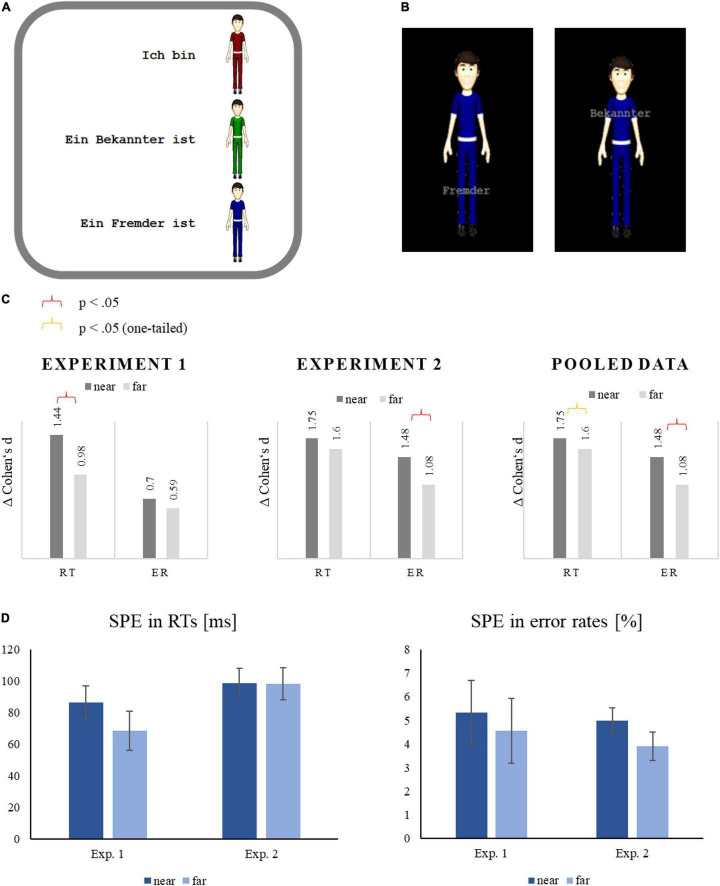
Examples of the presentation of the avatars in the experiments (not drawn to scale) as well as the results: Note that **(A,B)** show the original stimuli used. Thus, the language is German. Translated into English **(A)** reads “I am [red avatar],” “An acquaintance is [green avatar],” and “A stranger is [blue avatar].” In **(B)** the labels translate to “stranger” at the feet and “acquaintance” at the torso. **(A)** The slide, which was presented for 60 s to learn the avatar associations, **(B)** matching-task trials with near or far label in Experiment 1, and **(C)** the effect sizes in the near and far condition in both experiments and pooled above experiment. **(D)** The SPE in RTs and error rates depending on the experimental condition and the experiment.

We set out to investigate on how far an effect, which has been shown for participants themselves, can be transferred to an avatar. Specifically, the association of stimuli with the self is significantly stronger if the to-be-associated stimuli were perceived as being close to the self than when further away. The question was whether a similar effect would occur for an avatar, once this avatar was introduced as being self-relevant. Hence, we explored whether the location of stimuli relative to the self of an avatar influences performance overall and in particular the SPE. For this purpose, the distance between person-identifying labels and an avatar picture was varied in the matching task and the association of the labels with the self was tested in dependence on this distance. We postulate that the prioritization of self-associated stimuli—as indicated by the SPE—is larger when the associated labels are presented close to the upper torso of the avatar than when they are presented further away from it (Figure 1B).

### Methods

#### Participants and Design

A total of 33 participants (29 female and 4 male) with a median age of 21 years (ranging from 19 to 29 years except for one participant, who did not indicate their age) participated in the experiment, all having normal or corrected-to-normal vision. The mean error rate of one participant constituted an outlier (i.e., a far-out according to Tukey, 1977) and was therefore excluded before data analyses, resulting in a sample size of *N* = 32. In terms of power considerations, note that the SPE in the matching task with visual stimuli was rather large in previous studies (e.g., *d*_*z*_ > 0.65 (Sui et al., 2012; Schäfer et al., 2015). Consequently, a medium-sized effect of *f* = 0.35, given a medium-sized correlation between repeated performance measures of *r* = 0.4 and an α-value of 0.05, would be detected with a power of 1–β = 0.95 in a sample of 30 participants (G*Power 3.1.3; Faul et al., 2007).

Although three independent variables make up the experimental design plan—distance (*near* vs. *far*), avatar identity (*self* vs. *stranger*), and label (*self* vs. *stranger*)—the critical comparison deals with the SPE depending on the distance parameter. Prioritization effects are usually analyzed in matching trials because matching and non-matching trials involve different processes and prioritization has most reliably been demonstrated in matching trials (Schäfer et al., 2017; S. 20; Sui et al., 2016; Schäfer and Frings, 2019). Consequently, the critical contrast comprised a one-factorial design with “distance” (*near* vs. *far*) as a within-subject factor and the SPE as the dependent variable.

#### Apparatus and Materials

The experiment was conducted using standard PCs with standard TFT monitors, German QWERTZ keyboards, and by using E-Prime 2.0 software. The words were written in Courier New and in gray on a black background or partially in front of the picture of an avatar. At the beginning of each trial, a fixation cross was presented at the center of the computer screen. To show the combinations, a label was presented either at 10% of display height above or below the center of the screen, depending on the distance condition (above in the near condition and below in the far condition, see Figure 1; except for the practice trials, see below). The avatar was always presented centrally. With a viewing distance of about 60 cm throughout the experiment, the labels were presented subtending 0.7° visual angle high and 2.0° to 6.1° width and the avatars 14.0° visual angle high and 4.6° width. We used the German words Ich [I] as the self-relevant label, the German word “Bekannter” [friend] as a familiar stimulus, and the German word “Fremder” [stranger] as a neutral stimulus. To present the avatars, relatively detailed pictures of avatars were used, which had already been used in previous studies (Mattan et al., 2016). These avatar pictures were colored either red, green, or blue (e.g., for a presentation of the avatars, see Figures 1A,B). The assignment of the red, green, or blue avatar with the labels (either self-relevant or one of the neutral labels) was balanced across participants following a Latin-square design.

#### Procedure

Participants were tested in a soundproofed room. Each participant signed informed consent, specified their gender (male or female), and indicated their age. After that, task instructions were briefly summarized by the experimenter and then presented in detail on the screen.

The experiment comprised three phases, which were separated by short instruction slides. During the association phase at the beginning of the experiment, participants were instructed to learn assignments of avatars and labels. Thereto, the assignments of the labels (i.e., I, friend, and stranger) and the three different avatars (Figure 1A) were presented on the screen for 60 s.

The participants next went through a practice phase of the matching task. Here (and also for the test phase), participants were told to place the index finger of the left hand on the S-key (non-matching response) and the index finger of the right hand on the L-key (matching response). Each trial started with a 500-ms presentation of a black screen, followed by a fixation cross for 500 ms. Then, an avatar-label combination was shown for 200 ms, followed by a black screen until the participant responded or 1,500 ms had elapsed. Participants’ task was to judge whether the displayed combination corresponded to one of the initially learned assignments or not. During this practice phase, the labels were presented centrally (in front of the avatar picture). Thus, the assignments were learned with a neutral label position. During the practice phase, participants received feedback on whether their response had been correct, wrong, or too slow; the feedback slide was presented for 2,000 ms.

After the practice trials, the participants were informed that the practice phase of the matching task was over and that test trials will follow. In this test phase, the task was again to judge whether each presented avatar-label pairing corresponded to the initially learned assignment. In contrast to the practice phase, here no feedback was given and the position of the labels varied according to the distance condition, above the center (i.e., in front of the torso of the avatar) in the near condition and below the center (i.e., in front of the legs of the avatar) in the far condition^1^.

The test phase consisted of 504 trials presented in random order. In detail, each label was presented in 168 trials, half of them matching and half of them non-matching pairings (resulting in 84 matching and 84 non-matching trials for each label). Note that, in the matching task, non-matching trials are only included to make the task a useful task and they were completely irrelevant to our hypotheses. At the end of the experiment, participants were debriefed and thanked for their participation.

### Results

#### Response Times

Only correct responses with RTs above 200 ms and below three interquartile ranges above the third quartile of the overall distribution of correct RTs (Tukey, 1977) were used for the RT analysis. Averaged across participants, 75.8% of all trials were selected for RT analysis; 14.3% of the trials were excluded because of erroneous responses; and 9.8% due to the RT outlier criteria. Mean RTs and error rates in all conditions are shown in Table 1.

**TABLE 1:** RTs (in ms) and error rates (in %) of Experiment 1 as a function of distance, stimulus association, and match (standard deviations in parentheses).

		rTs		Error rates	
Distance	Stimulus association	Matching	Non-matching	Matching	Non-matching
*Near*	*Self*	550 (54)	627 (82)	5.3 (4.3)	5.5 (6.3)
	*Friend*	637 (100)	684 (107)	5.7 (4.6)	7.1 (6.0)
	*Stranger*	636 (97)	670 (97)	4.6 (4.6)	6.3 (4.7)
*Far*	*Self*	576 (50)	647 (77)	5.4 (4.9)	5.5 (4.7)
	*Friend*	644 (98)	697 (112)	6.6 (4.7)	7.7 (5.6)
	*Stranger*	644 (86)	692 (122)	5.1 (4.1)	7.2 (5.6)

The SPE is indicated by faster responses in matching trials with the self-associated stimulus (i.e., the stimulus associated with the label “I”) compared to responses in matching trials with one of the other-associated stimuli (i.e., the stimuli associated with the label “friend” or “stranger”; for a definition, see, e.g., Sui et al., 2012). Thus, we computed the SPE as this difference for each distance condition and compared the two effects. Either with the label in front of the torso and as well as with the label in front of the legs, a significant SPE was found, *t*(31) = 8.15, *p* < 0.001, *d* = 1.44 and *t*(31) = 5.57, *p* < 0.001, *d* = 0.98, respectively. Importantly, thereby supporting our hypothesis, the SPE was significantly larger when the label was presented in front of the torso—that is, near to the location of the self—than when it was presented in front of the legs—that is, further away from the location of the self, *t*(31) = 2.69, *p* = 0.011, *d* = 0.58 (Figure 1C).

#### Error Rates

Comparable to the RT analysis, an SPE in error rates was computed as the difference between the error rate in matching trials with the self-associated stimulus (i.e., the stimulus associated with the label “I”) and the averaged error rate in matching trials with the other-associated stimuli. The resulting SPEs were significant in the two distance conditions, *t*(31) = 3.94, *p* < 0.001, *d* = 0.70, in the near condition and, *t*(31) = 3.32, *p* = 0.002, *d* = 0.59, in the far condition. Moreover, the SPE was descriptively larger in trials with the label in front of the torso than in trials with the label in front of the legs, but this difference was not significant, *t*(31) = 1.55, *p* = 0.130, *d* = 0.24. Thus, the accuracy analysis revealed no significant effects but showed that there was no accuracy-speed trade-off.

### Discussion

Experiment 1 shows that the SPE was significantly larger when the label was presented in front of the torso—that is, near to the location of the self—than when it was presented in front of the legs—that is, further away from the location of the self. Hence, when the association label was presented in front of the torso of the avatar, identification with this avatar—and therefore prioritization of the self-associated avatar against the other avatars—seemed to be strong. However, it remains unclear whether this effect is robust across participants and can be replicated. Further, Experiment 1 includes a perspective-taking task block that is not part of the ordinary SPE paradigm. Consequently, Experiment 2 was conducted to test the robustness of the effect.

## Experiment 2

However, it remains unclear whether this effect is robust across participants and can be replicated. Further, as Experiment 1 included a hypothesis-irrelevant perspective-taking task, the study was replicated without this task to check whether a similar distance effect like in Experiment 1 will occur. Consequently, Experiment 2 was conducted to test the robustness of the effect. We postulate the same effect to occur as in Experiment 1: a significant influence of the distance from the labels to the hypothesized location of the self in the avatar indicated by a larger SPE when the associated labels are presented close to the upper torso of the avatar than when they are presented further away from it.

### Methods

#### Participants and Design

The data of 39 participants were collected (29 female and 10 male) with a median age of 21 years (ranging from 18 to 29 years), all having normal or corrected-to-normal vision. The sample of Experiment 2 was comparable to Experiment 1 and thus experimental power is held to the same standard across experiments. Further, although this experiment was carried out by the same group of experimenters, the participants did not overlap (i.e., a participant that was part of the previous study was not allowed to participate again), which potentially allows for direct replication of our findings. The mean error rate of three participants constituted outliers (i.e., far outs according to Tukey, 1977) and was therefore excluded before data analyses, resulting in a sample size of *N* = 36. Like in Experiment 1, the design was a one-factorial design with “distance” (*near* vs. *far*) as a within-subject factor.

#### Apparatus and Materials

The apparatus and material were the same as in Experiment 1 except for the following details. First, the labels were presented in front of a small gray box to potentially increase the readability of the labels. Second, male as well as female avatars were used depending on the participants’ sex as indicated at the beginning of the experiment to potentially increase the identification. For an example of a matching-task trial in Experiment 2 in comparison to Experiment 1 as well as for a picture of a female version of an avatar, see the Appendix.

#### Procedure

The procedure of Experiment 2 was completely the same as in Experiment 1 except that the block of the (hypothesis-irrelevant) perspective-taking task was no longer part of the study.

### Results

#### Response Times

Only correct responses with RTs above 200 ms and below three interquartile ranges above the third quartile of the overall distribution of correct RTs (Tukey, 1977) were used for the RT analysis. Averaged across participants, 74.5% of all trials were selected for RT analysis; 13.9% of the trials were excluded because of erroneous responses; and 11.6% due to the RT outlier criteria. Mean RTs and error rates in all conditions are shown in Table 2.

**TABLE 2:** RTs (in ms) and error rates (in %) of Experiment 2 as a function of distance, stimulus association, and match (standard deviations in parentheses).

		RTs		Error rates	
Distance	Stimulus association	Matching	Non-matching	Matching	Non-matching
*Near*	*Self*	519 (64)	598 (92)	1.9 (2.2)	5.3 (4.3)
	*Friend*	605 (94)	668 (114)	6.3 (4.9)	5.7 (4.6)
	*Stranger*	630 (107)	661 (91)	9.3 (5.2)	5.6 (4.6)
*Far*	*Self*	534 (66)	604 (95)	3.1 (6.0)	5.4 (4.9)
	*Friend*	620 (86)	668 (101)	6.0 (4.3)	6.6 (4.7)
	*Stranger*	645 (112)	666 (98)	9.5 (6.5)	5.1 (4.1)

Completely in line with the analyses in Experiment 1, the SPE was computed by the difference between RTs in matching trials with the self-associated stimulus (i.e., the stimulus associated with the label “I”) and RTs in matching trials with the other-associated stimuli (i.e., the stimuli associated with the label “friend” or “stranger”). As in Experiment 1, a significant SPE was found with the label in front of the torso and as well as with the label in front of the legs of the avatar, *t*(35) = 10.51, *p* < 0.001, *d* = 1.75 and *t*(31) = 9.62, *p* < 0.001, *d* = 1.60, respectively. However, the SPE in RTs did not differ at all between the two distance conditions, *t* < 1 (Figure 1C).

#### Error Rates

Comparable to the RT analysis, an SPE in error rates was computed as the difference between the error rate in matching trials with the self-associated stimulus (i.e., the stimulus associated with the label “I”) and the averaged error rate in matching trials with the other-associated stimuli. The resulting SPEs were significant in the two distance conditions: *t*(35) = 8.87, *p* < 0.001, *d* = 1.48 in the near condition and *t*(31) = 6.50, *p* < 0.001, *d* = 1.08 in the far condition. Remarkably, the SPE in error rates was significantly larger in trials with the label in front of the torso than in trials with the label in front of the legs, *t*(31) = 2.39, *p* = 0.022, *d* = 0.40.

### Comparison of the Experiments

To investigate on how far the two experiments reveal different or rather similar data patterns, the data of both experiments were put in one analysis with the factor “experiment” as a between-subject factor. Two two-factorial ANOVAs with the factors distance (*near* vs. *far*) and experiment (*Experiment 1* vs. *Experiment 2*) were conducted with either the SPE in RTs or the SPE in error rates as the dependent variable. All those participants whose performance was far out scores in the separate experiment analyses (one participant from Exp. 1 and three from Exp. 2) were excluded before analysis.

The ANOVA with SPE in RTs revealed a non-significant main effect of distance, *F*(1,66) = 3.20, *p* = 0.078, η_*p*_^2^ = 0.046. However, given the directional hypothesis and the results of Experiment 1, we see this one-sided significant results as tentative evidence to support the hypothesis. The main effect of the experiment was not significant, *F*(1,66) = 2.22, *p* = 0.141, indicating that SPEs did not vary between the groups. The interaction of both factors was also not significant, indicating only a tendency of a difference between the effects of distance in both experiments. The ANOVA with SPE in error rates, however, revealed a significant main effect of distance, *F*(1,66) = 7.64, *p* = 0.007, η_*p*_^2^ = 0.104, suggesting again larger SPEs in the near conditions than in the far conditions. Neither the main effect of the experiment nor the interaction of distance and experiment were significant, both *F*s < 1^2^, indicating that the SPE did not vary between the groups, and, more importantly, that the effect of distance did not differ at all between the two experiments.

## General Discussion

We investigated whether individuals can form a strong self-association with a previously neutral human avatar. In a simple matching paradigm, we investigated the performance advantage for stimuli presented close to the avatar, as this location represents the subjective location of the self in humans.

Two key results were found. First, stimuli presented close to the perceived location of the self are processed more efficiently. Second, the SPE was modulated by location as well. This indicates that self-relevant stimuli, that are presented close to the self, receive an additional boost. Overall, only 13 participants did not show an increased SPE in the near- compared to the far-condition in either RTs or error rates. Although some interindividual parameters seem to affect inter-individual variance, the effect is stable across experiments and individuals. The results give further insight into how people perceive themselves and representations of themselves in the outside world. The results further provide support for the idea that individuals can form strong self-association with an avatar and somewhat transfer themself into the avatar. The results are clear and replicated in two independent studies. Further, the present results suggest there to be an effect gradient across the avatar, with increased self-prioritization effects for stimuli presented as the avatar self-center (for congruent results see Schäfer et al., 2019).

The results can be compared and contrasted with Schäfer et al. (2019). In their study, the authors had participants associate a set of vibrations on the forearm with certain labels such as “stranger” or “self.” Thus, the vibrations were either physically closer to the participants’ ego-center or further away. In contrast, in the present study, a self-translation into an avatar needed to happen first. Although both studies show an effect of distance to the ego-center, the effect in the present study is smaller compared to Schäfer et al. (2019).

In addition, there is a boundary condition to be considered when interpreting the results and transferring them to real-life applications. It is important to note that the present results apply to avatars that have a clear ego center. The present study dealt with avatars resembling humans and it may be conjectured that they apply to all humanoid characters. However, it remains a topic of further research whether the present results may transfer when an avatar does not have a clear ego-center and does not correspond to any real-world being. Similarly, it needs to be tested whether the results may apply to games in which the avatar or at least its ego-center is not visible (e.g., first-person perspective games).

Another limitation to the present study is that we made use of static, 2D images, which is not reflective of a lot of modern games, in which animated avatars are often used. Although we think that the results may translate to more vivid environments, that is up for future research to determine. Nevertheless, a recent study using animated 3D avatars suggests that at least avatar identification, as measured by the avatar identification scale (Van Looy et al., 2012), is connected to the SPE and predictive of avatar-related performance in the task (Friehs et al., 2022).

Further, while the present study and its results are discussed in the context of games and gamification, it is crucial to note that the task used in this study does not contain many game-like elements (e.g., defined failure states, scoring systems). While gamification and games, in general, may be a useful research tools, they are not without risk, because they can change task performance in an undesired direction (Hawkins et al., 2013). For example, adding a simple scoring or reward system creates a motivational pull that can interact and interfere with the to-be-measured variable and change behavior (Bogacz et al., 2010; Hinvest and Anderson, 2010; Hickey et al., 2015). A reward can even capture attention when it is counterproductive to the task performance, which might make simple reward elements, for example, not always suitable for all gamification purposes (Le Pelley et al., 2015). Consequently, gamification must be approached in a stepwise manner. In this specific case, the SPE first needed to be established in the lab (Sui et al., 2012; Schäfer et al., 2015) and extended toward non-visual domains (Schäfer et al., 2020) before it can be transferred into applied settings using setups such as the present studies (see also Friehs et al., 2022).

### Implications

The evidence generated in the present study has clear implications for game designers. To reiterate, the results show, on the one hand, that individuals can transfer themself into an avatar and, on the other hand, that stimuli presented close to the self-center are processed more efficiently (especially when they are self-relevant). The present results have implications for all technology that aims to represent an individual digitally and present the individual with information. This may be best exemplified by the use of avatars in games, specifically the results have implications for the design of user-interfaces, fostering immersion and multiplayer gaming. First, user-interface may find these results beneficial, as they show that it has a significant impact on where information is displayed. This is especially relevant when designers have to decide whether to display information close to the character or at the edges of the screen. Against the background of the results, we argue that crucial information, which requires a quick reaction, should be displayed close to the self of the player’s avatar, whereas information that does not require a fast response can be displayed peripherally. This recommendation may also apply to gamified cognitive-psychological tasks that aim to measure human performance, on the condition that the task uses an avatar. For example, (Friehs et al., 2020a,b) developed a game-like version of the stop-signal task that utilizes an avatar. Further, increased identification with the avatar increased the performance and training effects of the task (Friehs et al., 2022). However, with all that being said, the paradigm utilized in the present study is not directly transferable to games as the visual fidelity and the in-game dynamic are vastly different. Thus, future research needs to determine how well the effect transfers to actual game environments. For example, it may be also possible that important information close to the participant may be distracting and detrimental to performance; for example, when a pop-up appears suddenly or obfuscates other information.

Second, one might also assume that displaying self-relevant information close to the avatar increases not only performance but also potentially the subjective identification with the avatar and the immersion (although this should be put to a direct test in further research). A good example of how the location of the information display can increase identification and immersion is the critically acclaimed video game series Dead Space by Electronic Arts. In the games, the protagonist is mostly viewed from behind and crucial information such as health points are integrated into the upper torso armor. We assume that this decision would lead not only to faster and more accurate reactions to the loss of health, for example, but also to increased immersion (as compared to a fictional Dead Space game version, which displayed the health bar at the bottom of the screen). However, we have no data to support this and this could be an avenue for future research to explore.

Third, the present results have a clear implication for multiplayer game design or games that support multiple (similar looking) characters in general. Since self-relevant information presented at a location close to the self receives an additional processing boost, we recommend choosing this location to display information that is relevant to that avatar and player.

Based on these, we like to highlight another potential impact of this research: an impact within the context of competitive gaming and e-sports. E-sports has been on the rise with e-sports college scholarships (e.g., University of California, Irvine), professional e-sports teams, gaming celebrity culture, and prize money (Newzoo, 2020), with winners of large e-sports tournaments winning more money than winners of the Wimbledon tennis championship or the Masters golf championship. One such competitively played game is World of Warcraft by Blizzard Entertainment. It is one of the largest and longest-standing Massively Multiplayer Online Role-Playing Games with a vibrant player base. Whenever a new expansion for this game is released, a race to overcome the newest content takes off. In this Race to World First, the competitive gamers will try to garner every possible advantage they can get, which also includes the modification of their interface to process most information as possible, as effectively as possible. Players and designers can learn from studies like the present one to further improve gaming experience and performance. There is also evidence of people already benefiting from the effect demonstrated in the present study without conscious knowledge of it. For example, many players in World of Warcraft and similar games tend to move their health bars close to the head of the avatar and their interface tends to focus around the center of their avatar. Thus, the SPE in combination with the heightened performance for stimuli presented close to the avatar’s self may be seen as a potential performance enhancement tool. Although there are other potential performance enhancers to be used (e.g., transcranial direct current stimulation or the intake of performance-enhancing supplements like Tyrosine), the optimization of UIs is non-problematic from an ethics and safety standpoint (e.g., Friehs et al., 2019, 2020b,^2021^; Frings et al., 2019).

## Conclusion

The present results from two independent studies show that the individuals can transfer their self into a previously neutral avatar. This conclusion is supported by the result that shows information that is being presented close to the ego-center of the avatar is processed more efficiently (i.e., with faster responses and lower error rates). In detail, the benefit for information displayed close to the avatar ego-center across both studies was ∼18 ms and 1.8 error %. As a comparison, a performance increase due to non-invasive brain stimulation in a game-based task may be around 20 ms (Friehs et al., 2020b) or 15 ms in a basketball-specific task (Friehs et al., 2019). This can also be seen when effect sizes are taken into account. Across experiments, Cohen’s *d* for the near condition for RT is 1.75 versus 1.6 in the far condition and 1.48 versus 1.08 for error rates, respectively. Notably, the effect size difference between distance conditions is significant (0.15 in RTs and 0.4 in error rates across experiments). This performance advantage, although small it may seem, can be critical in all situations where a quick and accurate response is key; such as video-game designers and players alike that aim to increase game performance. Further work is needed to determine whether this way of presenting information can also increase transportation and immersion in addition to performance.

## Data Availability Statement

The datasets presented in this study can be found in online repositories. The names of the repository/repositories and accession number(s) can be found below: http://dx.doi.org/10.23668/psycharchives.2365.

## Ethics Statement

Ethical review and approval was not required for the study on human participants in accordance with the local legislation and institutional requirements. The patients/participants provided their written informed consent to participate in this study.

## Author Contributions

SS supervised the data collection and was responsible for data analysis. MF provided the first draft of the manuscript and was responsible for writing. CF provided administrative support and supervision. All authors contributed to the article and approved the submitted version.

## Conflict of Interest

The authors declare that the research was conducted in the absence of any commercial or financial relationships that could be construed as a potential conflict of interest.

## Publisher’s Note

All claims expressed in this article are solely those of the authors and do not necessarily represent those of their affiliated organizations, or those of the publisher, the editors and the reviewers. Any product that may be evaluated in this article, or claim that may be made by its manufacturer, is not guaranteed or endorsed by the publisher.

## Footnotes

1 Note that the experiment also included (between practice phase and test phase of the matching task) one block of a perspective-taking task, which was, however, completely irrelevant for the hypothesis.

2 The value is calculated by dividing the explained variance by the unexplained variance. Thus, an *F*-value smaller than 1 is indicative of a statistically insignificant result. To keep the manuscript concise and brief we excluded a detailed statistical description for these kinds of results.

## Appendix

Avatar and label presentation in Experiment 1 (left picture) and Experiment 2 (right picture). Depicted are examples from the learning phase of the matching task, in which the labels were presented in the center.

Example of a female avatar as they were used in Experiment 2.
